# Facts Relative to the Prevention of Hydrophobia

**Published:** 1792

**Authors:** Jesse Foot

**Affiliations:** Surgeon in London.


### V. *Facts relative to the Prevention of Hydrophobia. Communicated in a Letter to Dr.* Simmons *by Mr.* Jeſſe Foot, *Surgeon in London.*

I HAVE the honour of preſenting to you ſome facts relative to the prevention of hydrophobia, which I wiſh to ſee publiſhed as an excitement to a ſimilar mode of treatment in other inſtances of the ſame kind; being thoroughly perſuaded that exciſion of the bitten part is the only remedy that ought to be truſted to in ſuch caſes.

The facts now communicated to you are in addition to two other ſtriking and ſucceſsful inſtances of the ſame ſort formerly given to the Public, by me, in an Eſſay * on this ſubject, to which I beg leave to refer you.

### CASE I.

Two years and a half ſince a young lady was bitten in the heel by her own lap dog, ſo as to produce a bleeding; the dog died mad the next day. I was preſent when the bitten parts were cut out, and the lady has remained well. The operation was performed three days after the accident.

### CASE II.

Sixteen months ago a ſtrange dog came into the yard of Robert Jackſon, Eſq., near Tewkſbury in Glouceſterſhire, and bit two dogs: they were both bathed in the ſea, and to both the Ormſkirk medicine was given. They both died mad; but one of them, having got looſe during his madneſs, bit a puppy that he had formerly been very fond of: the bitten part of the

* An Eſſay on the Bite of a Mad Dog. 8vo. London, 1788.

puppy was cut out directly, and the puppy continues well.

### CASES III. AND IV.

For these two cases I am indebted to Mr. John Capon Weeks, Surgeon at Rochester, and I shall give them to you in his own words, extracted from his letter to me on this subject, dated March 12th, 1791.

" George Cobb, a servant of Commodore " Pasley, was bit by a dog on the 29th of " November, 1789. I saw him immediately " after the bite. The bite was on the right " cheek, and the inside of the upper lip; but " I could not persuade him to have the parts " that were bitten extirpated. The Ormskirk " medicine was made use of, and the Birling " medicine was also given. The lad continued " very well for ten days after the bite, and the " wounds healed; but on the 10th of December, " symptoms of hydrophobia appeared. " The Birling medicine was now again administered; but by two o'clock of the morning " of the 12th of December the patient appeared quite exhausted by the violence of " the symptoms, became quiet, and died in " about half an hour after.

 " Richard

" Richard Braham was bit by the ſame dog " in about a quarter of an hour after the bite " of the unfortunate George Cobb. I ſaw this " lad about an hour after the bite. The parts " bitten were the upper lip, the under part of " the lower jaw, and the little finger of the " right hand, all of which bled in conſequence " of the bites. The bitten parts were all ex- " tirpated. The wounds were dreſſed with a " mercurial digeſtive, and healed in about ſix- " teen days. This patient has experienced no " ill effects from the bite, and is now in good " health."

*Dean Street, Soho,*

April 7, 1792.

